# Time-Resolved Transcriptional Profiling of Epithelial Cells Infected by Intracellular *Acinetobacter baumannii*

**DOI:** 10.3390/microorganisms9020354

**Published:** 2021-02-11

**Authors:** Nuria Crua Asensio, Javier Macho Rendón, Marc Torrent Burgas

**Affiliations:** Systems Biology of Infection Laboratory, Department of Biochemistry and Molecular Biology, Universitat Autònoma de Barcelona, 08193 Cerdanyola del Vallès, Spain; nuria.crua@uab.cat (N.C.A.); javier.macho@uab.cat (J.M.R.)

**Keywords:** *Acinetobacter baumannii*, infection, intracellular, transcriptional profile

## Abstract

The rise in the number of antibiotic-resistant bacteria has become a serious threat to health, making it important to identify, characterize and optimize new molecules to help us to overcome the infections they cause. It is well known that *Acinetobacter baumannii* has a significant capacity to evade the actions of antibacterial drugs, leading to its emergence as one of the bacteria responsible for hospital and community-acquired infections. Nonetheless, how this pathogen infects and survives inside the host cell is unclear. In this study, we analyze the time-resolved transcriptional profile changes observed in human epithelial HeLa cells after infection by *A. baumannii*, demonstrating how it survives in host cells and starts to replicate 4 h post infection. These findings were achieved by sequencing RNA to obtain a set of Differentially Expressed Genes (DEGs) to understand how bacteria alter the host cells’ environment for their own benefit. We also determine common features observed in this set of genes and identify the protein–protein networks that reveal highly-interacted proteins. The combination of these findings paves the way for the discovery of new antimicrobial candidates for the treatment of multidrug-resistant bacteria.

## 1. Introduction

The growing resistance of pathogens is a matter of real concern today. Indeed, several authors have described drug-resistant bacteria for which few, or no, treatments are available [1]. The failure to identify new antibiotics or molecules to fight these bacteria is becoming a threat to health systems across the globe [2]. In this context, understanding how pathogen bacteria can survive inside a host is therefore crucial [3].

*A. baumannii* is considered to be one of the most important bacteria pathogens. These are commonly referred to as ESKAPE organisms (*Enterococcus faecium*, *Staphylococcus aureus*, *Klebsiella pneumoniae*, *Acinetobacter baumannii*, *Pseudomonas aeruginosa,* and *Enterobacter*), and have the capacity to evade the actions of antibacterial drugs [4] and survive prolonged and harsh treatments. Specifically, thanks to a small genomic region of 86 kilobases containing 45 resistance genes, *A. baumannii* has become extremely resistant to several antimicrobial molecules [5,6].

For all the above mentioned reasons, *A. baumannii* has emerged as one of the main pathogens responsible for hospital- and community-acquired infections [7,8], and only few antibiotics can eradicate the infections it causes [7]. Some strains have become resistant even to carbapenems and polymyxins, meaning that combination therapy is the final treatment option available in such cases [5].

Compared to other microorganisms, like *Pseudomonas aeruginosa*, *Yersinia enterocolitica,* and *Helicobacter pylori* [9,10,11], *A. baumannii* is commonly regarded as a low-virulence pathogen [12,13]. Nevertheless, it can persist in the body for a prolonged period, and it is also known to invade human lung, laryngeal, and cervical epithelial host cells [13,14]. Consequently, *A. baumannii* has gradually come to be regarded as an important human pathogen in the hospital environment [7,8].

The adherence of *A. baumannii* to host cells has been the subject of a number of studies, which illustrate how several of its proteins are involved in this process. In particular, it uses a zipper-like mechanism that has also been described in several other bacteria [15,16]. This mechanism is receptor mediated; thus, it requires the direct interaction of bacterial ligands with the host’s cell-surface receptors and involves local cytoskeletal rearrangement at the invasion site [14,15]. OmpA, which is an abundant surface protein essential for isogenic cell invasion, is a key driver of this process. Indeed, *A. baumannii* mutants that are defective in OmpA are unable to infect the host [17]. Other proteins, like Phospholipase D (PLD) and the trimeric autotransporter adhesin Ata, have also been described as key effectors of the invasion of eukaryotic host cells by *A. baumannii* [18,19].

How *A. baumannii* survives inside host cells is less clear. Chul Hee Choi et al. have suggested that it lives within membrane-bound vacuoles in the cytoplasm, similar to other intracellular pathogens, e.g., *Neisseria, Listeria, Salmonella* and *Yersinia* [15]. However, studies of the intracellular lifestyle of *A. baumannii* are limited in number, with more research required to understand the pathogenicity of this microbe.

In this study, we analyzed the host response to an infection by *A. baumannii*. In particular, we performed time-resolved RNA-sequencing in an attempt to examine how this infection impacts the host expression response. Such an approach is used to quantify RNA in time-series frameworks and can provide information on the mechanisms used by a host to defend itself against an infection. Understanding the pathogen effect on hosts is vital, particularly to the development of strategies and therapeutic tools with which to control multi-resistant bacteria like *A. baumannii*.

## 2. Materials and Methods

### 2.1. Bacterial Cell Culture

The *A. baumannii* (Bouvet and Grimont 1986 strain, designation NCTC 7844) used in our study was purchased from Colección Española de Cultivos Tipo (CECT 452); human epithelial cells (HeLa, ATCC^®^ CCL-2™) taken from a human cervix epithelium were bought from ATTC (Manassas, VA, USA). The bacteria strain was transformed with pSC101 plasmid (Addgene) labeled with a timer-fluorescent protein.

### 2.2. Cell Culture and Infection

Our cells were grown in a Minimum Essential Medium (MEM, Gibco, Waltham, MA, USA), supplemented with 10% heat-inactivated Fetal Bovine Serum (FBS, Gibco), in a humidified incubator with 5% CO_2_ at 37 °C. The HeLa cells were then routinely passaged every 3 or 4 days. For seeding purposes, the cells were rinsed with Phosphate Buffer Saline (PBS) and incubated with a 3 mL Trypsin-EDTA solution (Gibco, Waltham, MA) for 5 min until the cell layer was seen under microscope observation to be completely dispersed. Six mL of MEM was then added to inactivate the trypsin-EDTA. The samples were subsequently centrifuged for 5 min at 1000× *g* and sub-cultured at a ratio of 1.5 × 10^4^ cells/cm^2^.

Using a fresh MEM, the HeLa cells were collected and seeded into 24-well plates at a ratio of 3 × 10^4^ cells/mL and incubated for 48 h. Before infection, the *A. baumannii* cells were cultured overnight at 37 °C, with agitation at 250 rpm. The bacteria were diluted at a ratio of 1/1000 in a fresh medium and grown to OD_600 nm_ = 0.2. The HeLa cells were infected with *A*. *baumannii* at a multiplicity of infection (MOI) of 1:50. Three biological replicates were used for each sample at 2, 4 and 6 hours post-infection (HPI). After adding bacteria, the plates were centrifuged for 5 min at 250× *g* to improve the contact between the bacteria and cells. After 1 h of infection, the cells were washed twice with PBS and the medium was replaced with a version supplemented with 100 μg/mL gentamicin to kill the extracellular bacteria.

### 2.3. Flow Cytometry

The infected cells were washed 5 times with 1 mL of 1% PBS at the defined post-infection time-points. We then added 100 μL of a Trypsin-EDTA solution. After incubation for 5 min, we added 1 mL of MEM to inactivate the Trypsin-EDTA solution and transferred all the samples into a microtube. We then centrifuged them for 5 min at 7500× *g* and resuspended them in 1 mL of 1% PBS. The samples were analyzed with a flow cell cytometer (FACSCanto, BD Biosciences, San Jose, CA, USA).

### 2.4. Fluorescent Microscopy

At 2, 4 and 6 HPI, the samples were cleaned 5 times with 1 mL of 1% PBS and fixed with 1 mL of 4% Formaldehyde. After 15 min of incubation at room temperature, the samples were: cleaned 3 times with 1% PBS, to which we added 100 μL of DAPI (4’,6-Diamidino-2-Phenylindole, Dihydrochloride as per manufacturer’s protocol, Invitrogen); incubated for 5 min; and cleaned again 3 times with 1% PBS. We then added 100 μL of Cell Mask Deep Red Stain (Invitrogen, Waltham, MA, USA) and cleaned the samples again 3 times with 1% PBS. Images were subsequently obtained using an EVOS M5000 fluorescent microscope (Life Technologies, Waltham, MA, USA).

### 2.5. RNA Isolation

The infected cells were cleaned 5 times at 2, 4 and 6 HPI with 1 mL of 1% PBS. After 5 min of incubation, the samples were mixed with 100 μL of a Trypsin-EDTA solution, which was inactivated with 1 mL of a MEM. We transferred all the samples into microtubes, centrifuged them for 5 min at 7500× *g*, and resuspended them with TRIzol Reagent (Invitrogen). RNA extraction was conducted following the manufacturer’s protocol.

### 2.6. Read Mapping and Differential Expression Analysis

We prepared a total of 24 RNA samples (comprising 3 biological replicates, representing 0, 2, 4 and 6 HPI). Sequencing was performed at the Centro Nacional de Análisis Genómico (CNAG) using a 2500 HiSeq platform. Quality control of the raw RNA-Seq reads was conducted using FastQC. The reads were mapped to the Homo sapiens reference genome GRCh38 (hg38) using the default settings of STAR (version 2.6.1b) [19]. Mapped data were transformed into gene-level counts using human Gencode annotations (https://www.gencodegenes.org/human/; accessed on 31 December 2020) and the FeatureCounts software (http://subread.sourceforge.net/; accessed on 31 December 2020) [20]. Using DESeq2 [21], a time-series differential expression analysis was carried out for each time-point by comparing the data obtained at 2, 4 and 6 HPI to a sample at 0 HPI (control conditions). Differentially Expressed Genes (DEGs) were defined using the following criteria: |log2-fold change| >= 1 and adjusted *p*-value <= 0.05. Sequencing files were deposited in the Gene Expression Omnibus (GEO) database under code GSE161833.

### 2.7. Clustering and Functional Enrichment Analysis

Venn diagrams were constructed to identify which DEGs were shared between 4 and 6 HPI. The resulting groups were employed to perform a functional enrichment analysis using the g:Profiler web tool [22]. Untranslated region (UTR) and Promoter analysis were performed using ShinyGO, using an FDR < 0.05 [23].

## 3. Results and Discussion

### 3.1. Acinetobacter baumannii Can Survive inside Epithelial Cells

**Figure 1:**
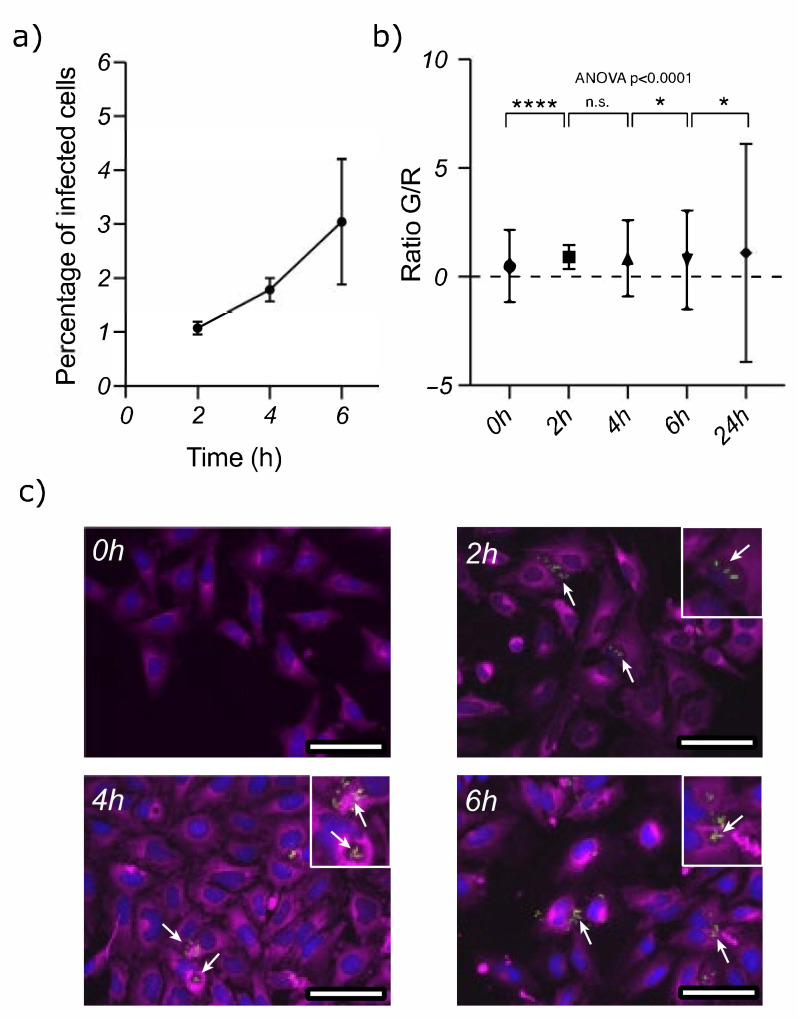
*A. baumannii* is able to survive intracellularly in HeLa cells. (**a**) This graph represents the *A. baumannii* infection rate in the HeLa cells; the X-axis relates to 3 time-points: 2, 4 and 6 hours post infection (HPI), and the Y-axis represents % of infection. The data show an increase in the number of infected cells at different HPI. (**b**) Flow cytometry of the cells infected with *A. baumannii* at the different HPI. *A. baumannii* carries a plasmid expressing the TIMER protein [24,25], enabling the tracking of intracellular-bacteria survival inside HeLa cells. The X-axis represents different time-points: 2 (in blue), 4 (in red) and 6 HPI (in green), and the Y-axis shows the green/red fluorescence ratio (G/R). A higher G/R ratio indicates that *A. baumannii* cells are replicating faster. (**c**) Fluorescence microscopy images (left to right) at 2, 4 and 6 HPI. The plasma membrane and nuclei of the HeLa cells were stained with CellMask (purple) and DAPI (in blue), respectively. The bacteria were detected using TIMER fluorescence. Bacteria foci details are shown as inserts in the top-right corner of each image. Scale bars represent 125 μm length. In all plots, standard error of the mean is represented by vertical bars. One-way ANOVA was used to determine whether there are any statistically significant differences between the means of two or more independent groups in the time series. Individual groups were compared using a two-tailed *t*-test: * *p* < 0.05, **** *p* < 0.0001, n.s. not significant.

In a first stage, we investigated whether *A. baumannii* is able to survive inside epithelial cells. To this end, we incubated HeLa cells with *A. baumannii* (MOI 50) for 2 h and then removed the extracellular bacteria using gentamicin. We quantified the surviving bacterial cells at 2, 4 and 6 HPI. Our results revealed that *A. baumannii* was able to colonize 0.3% of the HeLa cells and started to slowly replicate at 4 HPI, reaching 2.3% at 6 h (Figure 1a).

The *A. baumannii* cells were labeled with the TIMER protein to investigate whether they could grow intracellularly [24,25]. The fluorescence of TIMER is dependent on maturation and shifts from green to orange. When cells divide faster, the dilution of TIMER in the daughter cells remains fluorescent green. However, when cells do not divide, mature protein accumulates, and the fluorescence becomes orange–red. The bacterial cells prior to infection had a green/red fluorescence ratio of 0.5, increasing over time to 1.0. These changes show that cells start to divide slowly after infection, even after 6 h of incubation (Figure 1b). The small rise in the fluorescence ratio suggests that the intracellular bacteria growth was constrained due to a harsh intracellular environment. We also observed an increase in the fluorescence ratio’s standard deviation, meaning that the intracellular bacteria were highly heterogeneous and could replicate at different rates. Consequently, although the average replication in the population increased, there was a significant range in the metabolic rate of the bacteria cells that may have had an impact on the outcome of the infection. We used microscopy and the fluorescence of the TIMER protein to investigate the distribution of the bacteria inside the cells. These images show that at 2 HPI there were very few bacteria inside the host; they did, however, accumulate inside the HeLa cells over time, as identified previously via cell counting.

These results show that *A. baumannii* has the potential to invade, survive and replicate inside epithelial cells (Figure 1c). Other research groups have described the intracellular nature of *A. baumannii* and the presence of 1–2 adhering bacteria per infected cell. *A. baumannii* thus seems to have a low cellular-invasion potential. Indeed, only 2.3% of our HeLa cells were infected at 6 HPI, with an average of 1.33 bacteria per cell. This is consistent with other studies in the literature [15].

### 3.2. Acinetobacter baumannii Reorganizes the Cell Transcriptome after Intracellular Invasion

**Figure 2:**
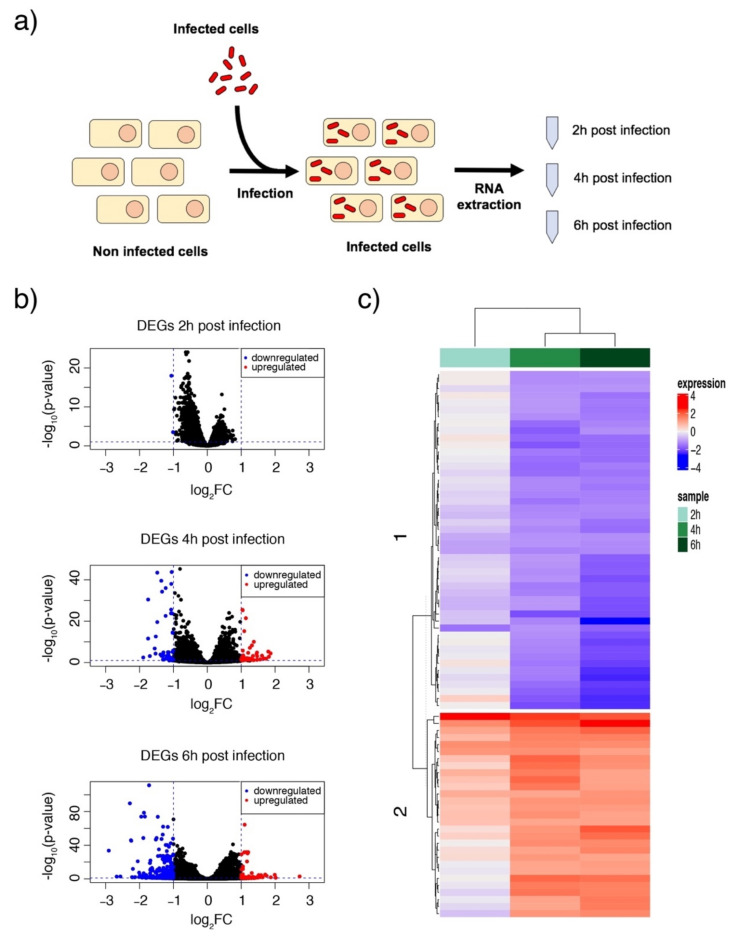
RNA sequencing and analysis of the expression profiles of Differentially Expressed Genes (DEGs). (**a**) HeLa cell infected with *A. baumannii* at three different infection time-points; RNA extraction was conducted at 2, 4 and 6 h after infection. (**b**) Volcano plots to portray the DEGs. DEGs at 2 h (top), 4 h (middle) and 6 h (bottom) after infection are represented: the X-axis indicates log_2_ (FC) and the Y-axis -log_10_ (*p*-value). The red dots represent upregulated genes (*p*-value < 0.05 and log_2_FC < −1) and the blue dots downregulated genes (*p*-value < 0.05 and log_2_FC > 1). This analysis revealed that the changes at 2 h were, globally, not significant compared to the position at 0 h. (**c**) Heatmap showing the relative expression levels for the DEGs (expression type: *p*-value < 0.05 and log_2_FC < −2/+ 2). Two main clusters were observed: downregulated genes (blue; cluster 1) and upregulated genes (red; cluster 2). Each column represents the expression level (log_2_ fold change) at different time-points: 2 h (light green), 4 h (green) and 6 h (dark green).

We also investigated the host response to an intracellular *A. baumannii* infection. Transcriptomic studies can provide useful information on underlying pathogenic mechanisms and interactions when following the course of an infection. Consequently, we infected HeLa cells with *A. baumannii* at an MOI of 50 and tracked it over time (2, 4 and 6 h). The host RNA was isolated and sequenced at every time-point. Non-infected HeLa cells were used as a control (Figure 2a). Consistent with our previous findings, we noted very few significant differences (fold change > 1 and adjusted *p*-value < 0.05) at 2 HPI, indicating that only few HeLa cells were infected intracellularly (Figure 2b). We are aware that this may limit our characterization of the early stages of infection, i.e., adhesion and the entry of the bacteria into the host cells, but we were nevertheless interested in how *A. baumannii* survives and proliferates therein. Moreover, a higher bacterial load would have caused extensive damage to the epithelial sheet, confounding the results. We only detected two genes that were differentially expressed: COL5A1 and IGF2R, involved in collagen metabolism and intracellular trafficking, respectively. Other studies have also consistently reported that COL5A1 and other members of the collagen superfamily are downregulated during the early stages of infection [26]. Reductions in collagen and other collagen precursors may suggest a loss of tissue tensile strength. The downregulation of IGF2R that we observed is also interesting, as this receptor is involved in the intracellular trafficking of lysosomal enzymes. Reduced levels of the IGF2 receptor may indicate an impaired lysosomal function that would help *A. baumannii* to survive intracellularly. Indeed, the persistence of *A. baumannii* was associated with less lysosome acidification [27].

**Figure 3:**
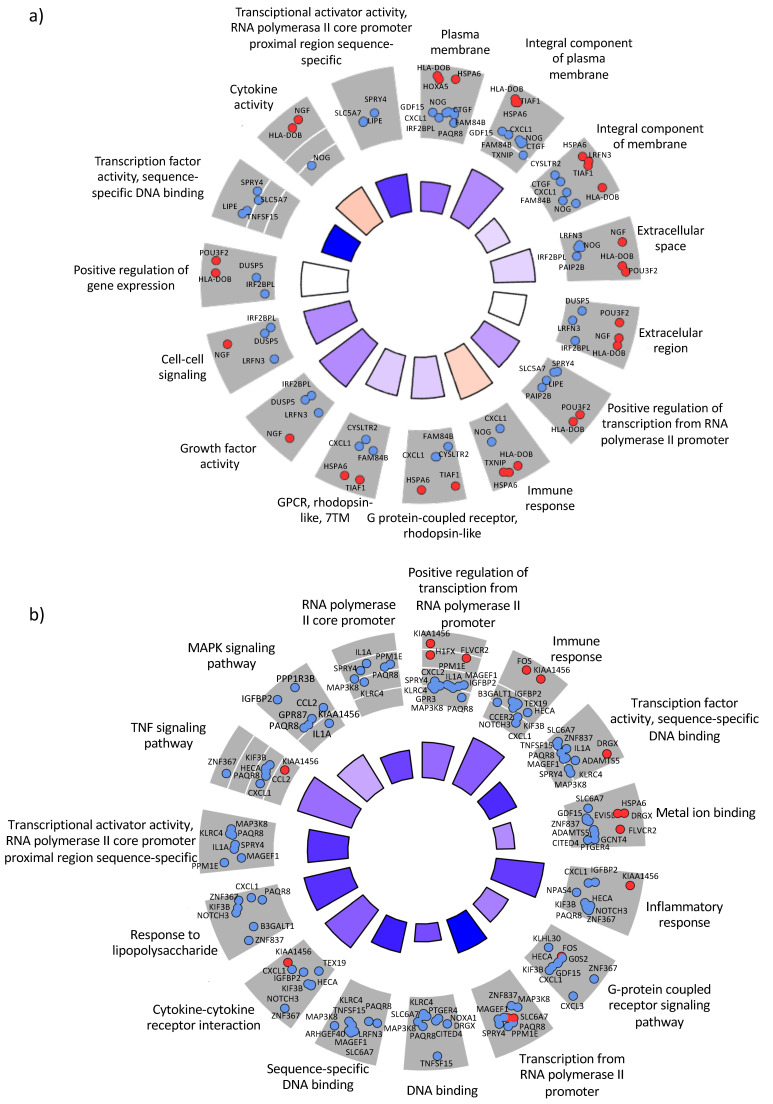

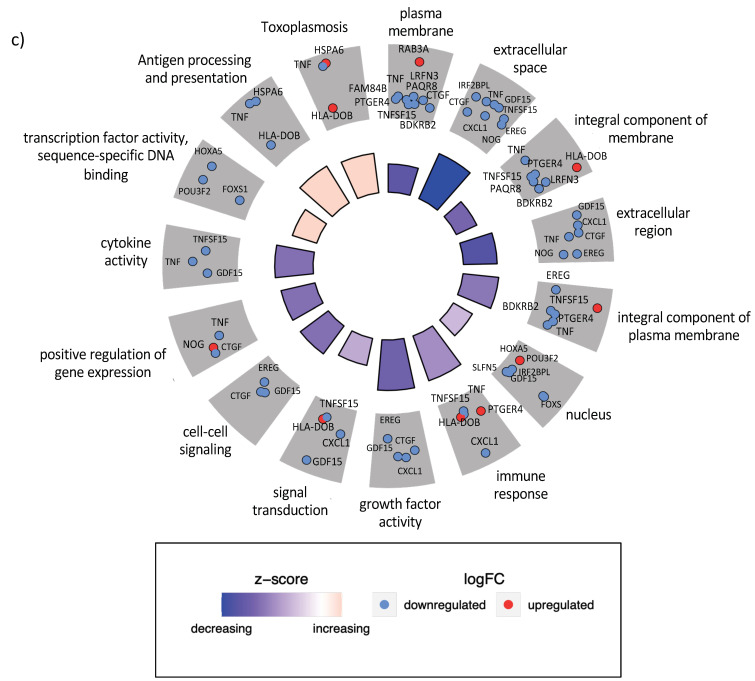
Gene Ontology (GO) clustering using chord diagrams for DEGs at three different time-points after infection. (**a**) Common DEGs at 4 h and 6h after infection, (**b**) DEGs exclusively found at 4 h and (**c**) DEGs exclusively found at 6 h. Red circles represent upregulated genes and blue downregulated genes. Overall GO differential expression z-score is also displayed as bars in the inner circle of the diagram. All GO terms and gene names are displayed in the diagrams.

As expected, the number of DEGs at 4 and 6 HPI increased to 136 and 464, respectively. At 4 h, more than 50% of the genes (75) were upregulated, whereas at 6 h more than 80% (390) were downregulated (Figure 2c). These results suggest a dynamic, time-dependent rearrangement of gene expression during intracellular replication. A Gene Ontology (GO) analysis of these findings identified that the host immune response and cytokine activity at 4 h is upregulated but downregulated at 6 h (Figure 3a). This change may represent how bacteria alter the host cell environment for their own benefit. In contrast, antigen processing and presentation pathways were always upregulated (Figure 3). A more detailed focus at 6 h suggested a significant downregulation of the inflammatory response, TNF, and MAPK signaling pathways (Figure 3c).

The HLA class II histocompatibility antigen was among the main genes upregulated at 4 h and is known to be induced in both professional and non-professional antigen-presenting cells. This is consistent with *A. baumannii* acting as an extracellular pathogen that would be engulfed and digested in lysosomes, with the resulting peptides loaded on to MHCII molecules. Other major upregulated genes are related to the stress response and the regulation of cell death. Among the most upregulated genes we detected were the TGFB1-induced anti-apoptotic factor 1 (TIAF1) and the DNA damage-inducible transcript 4 protein (DDIT4). On the one hand, TIAF1 controls the signaling of the TNF receptor and the overexpression of TIAF1 in epithelial and monocytic cells, thereby inducing apoptosis [28,29]. On the other, DDIT4 inhibits cell growth by repressing the activity of the mammalian target of the rapamycin complex 1 (mTORC1). The overexpression of DDIT4 has also been reported to trigger apoptosis in *Staphylococcus epidermis*, which is probably a protective mechanism to avoid replication inside the host and a defense against viral replication [30]. Finally, we also observed the upregulation of the Heat shock 70 kDa protein 6 (HSPA6), the RAS-related protein Rab-3A (RAB3A) and the Thioredoxin-interacting protein (TXNIP). Upregulation of these genes was a sustained reaction at all time-points, suggesting that these cell mechanisms are triggered in response to infection and are maintained until the bacteria are cleared from cells. These proteins may play a role in membrane trafficking. Host cell Rab GTPases mediate intracellular transport phagocytosis or endocytosis of bacterial pathogens. For example, Rab3A is a target for the *Pseudomonas aeruginosa* ExoS protein to control exocytosis [31]. In addition, the *Brucella* infection reduces the expression of TXNIP in order to promote its intracellular growth in macrophages, which it achieves by reducing the production of Nitric Oxide (NO) and Reactive Oxygen Species (ROS) [32].

On the downregulated side, we identified antiapoptotic molecules like Ubiquitin carboxyl-terminal hydrolase 27 (USP27X) and inflammatory markers such as the chemokine CXCL1, the tumor necrosis factor α (TNFα), and the prostaglandin E2 receptor PTGER4. Accordingly, it has been reported that, at 4 HPI, *A. baumannii* started to cause the reduction in the levels of pro-inflammatory mediators by secreting effectors that block innate immunity signaling [33]. Meanwhile, it will be seen later that inflammation was further downregulated at 6 HPI.

Most of the genes upregulated at 6 h have functions related to the immune response and endomembrane system. Most were likewise upregulated at 4 h, including RAB3A and DDIT4. However, we also identified that the oxidized low-density lipoprotein receptor 1 (ORL1) was similarly upregulated. This receptor mediates the recognition, internalization and degradation of oxidatively modified low-density lipoproteins, increasing the production of ROS and possibly acting synergistically with TXNIP.

As noted, most coding RNAs appeared to be downregulated at 6 h. However, despite many genes also being downregulated at 4 h, there was a decrease in the abundance of new transcripts. This was particularly the case for the chemokines CXCL2, CCL2 and CXCL3, interleukin IL1α, and the proepiregulin that links CXCL1 and PTGER4, which was already downregulated at 4 h. Moreover, kinase kinase 8 (MAP3K8), the mitogen-activated protein kinase, was also downregulated, contributing to decreased TNFα activation. We also detected a downregulation of cytoskeleton-related proteins, including: the Serum Response Element (SRE) involved in the transduction of mechanical signals from cytoplasmic actin; cytosolic carboxypeptidase 4 (AGBL1), which plays a part in the deglutamination of tubulin; the kinesin-like protein KIF3B, which is involved in microtubule sliding and translocation; and the protein phosphatase 1E (PPM1E), which has a role in inhibiting stress breakdowns of actin fibers.

In summary, we observed that cells respond to *A. baumannii’s* intracellular survival and replication by using different mechanisms to induce apoptosis. This strategy has repeatedly been described as an effective means for stopping the growth of intracellular microorganisms [34,35]. Moreover, cells increase the production of ROS to kill pathogens and downregulate the reorganizing cytoskeleton proteins, probably as a way to reduce microorganism uptake. We also observed a general decrease in proinflammatory cytokines and other factors. These findings make it tempting to speculate that *A. baumannii* suppresses the release of these molecules to block the activation of the immune system, which is a strategy that is also employed by other intracellular pathogens.

### 3.3. Regulation of Host Genes in Response to A. baumannii Infection

**Figure 4:**
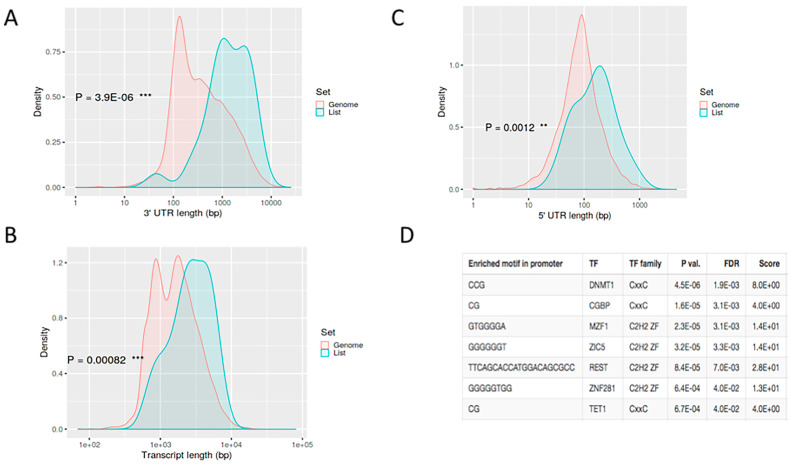
Identification of post-transcriptional regulation genes in *A.baumannii* infection. (**A**) 3′ untranslated region (UTR) and (**B**) 5′ UTR length analysis shows that downregulated genes have longer untranslated regions. (**C**) Transcript length analysis shows an increased transcript length for downregulated genes. (**D**) Analysis of motif enrichment in gene promoter regions suggests that several transcription factors may play a role in downregulating certain genes in response to *A. baumannii* infection. The explanation for , * and *** is as follows: *, ** and *** is as follows: * *p*-value < 0.01; ** *p*-value < 0.001; *** *p*-value < 0.0001

We used our gene expression analysis in an attempt to identify the possible post-transcriptional regulation of genes. We did not, however, detect any relevant trends in the upregulated genes, i.e., no genomic features that may explain coordinated regulation, but the downregulated genes had several features in common. A particularly interesting observation was that, in general, the downregulated transcripts had longer 5′ and 3′ untranslated regions (UTRs) (Figure 4A,B) and transcripts are also longer (Figure 4C). Unlike the coding region, both the 5′ and 3′ UTRs of mRNAs are enriched for Ribosome Binding Protein (RBP) sites [36]. Consequently, longer UTRs probably contain larger numbers of binding sites for RBPs, meaning that these genes are translated faster. This is compatible with a general repression of transcription after infection [37].

We were also able to relate several transcription factors (Figure 4D), including NFKB1, DNMT1 and CGBP, to gene downregulation. While it is well-known that NFKB1 is associated with infection and inflammation, the other two are zinc finger transcription factors that regulate the methylation of CpG islands. While DNMT1 methylates CpG residues, CGBP binds to unmethylated CpG motifs. Although this is only an indirect inference, it suggests the possible epigenetic regulation of infection. A detailed study of this issue is well beyond the scope of this paper, but it has been investigated by various researchers in the field, who have linked DNMT1 to viral infections [38] and uropathogenic *Escherichia coli* [39].

### 3.4. Gene Expression Changes Are Correlated at the Level of Protein–Protein Interactions

**Figure 5:**
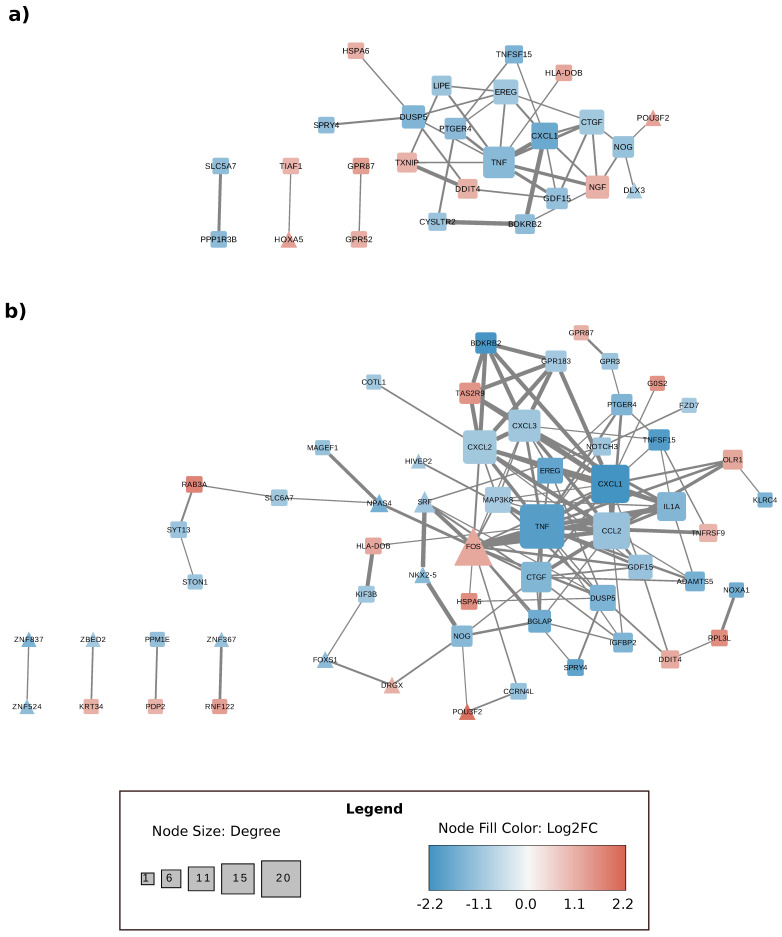
Cytoscape representation of the protein–protein interaction (PPI) network of our sorted DEGs. Representation of the PPI network at (**a**) 4 h and (**b**) 6 h. Network diagrams are useful for visualizing hub proteins and their immediate connections. The red nodes are proteins codified by upregulated genes and the blue nodes proteins codified by downregulated genes. The square nodes are generic proteins and the triangle nodes transcription factors. The connecting lines represent directional co-dependency in expression; the light to dark arrows show moderately-to-highly-interacted scores between the proteins (*p*-value < 0.05 and log_2_FC < −1/+1). The size of a node is proportional to the number of connecting lines involved.

Several studies suggest that protein-protein interactions are fundamental for the pathogens to infect the host [40,41]. Hence, we also examined whether genes found to be differentially regulated during infection can be connected at the protein level. We used the data stored in the String database to connect the proteins encoded in the DEGs. It was easy to observe that most of these genes were closely connected at 4 h (Figure 5a). For example, DUSP5, TNF, CTGF and CXCL1 appeared as hubs in our network, connecting with most of the DEGs. This was also the case at 6 h, when the clusters were even more compact (Figure 5b). These results suggest that transcriptional changes are also connected to protein–protein interactions (PPIs), meaning that there is a coordinated change in the transcription and interactome levels. Such evidence has been reported previously by our group in relation to uropathogenic *E. coli* [42,43]. Moreover, dense clusters with specific functions were seen if we expanded this network to include the first neighbors of the differentially regulated proteins (Figures S1 and S2, Supplementary Materials).

## 4. Conclusions

**Figure 6:**
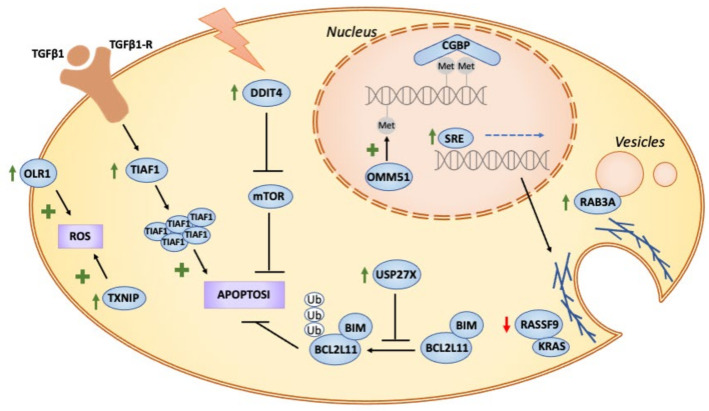
Schematic illustration of the proposed molecular mechanism of *A. baumannii* infection in mammalian cells. Based on our DEGs analysis we propose an increase in ROS production that may be due, in part, to the upregulation of OLR1 and TXNIP. Apoptosis is also activated by key genes, such as DDIT4, TIAF1 and BCL2L11. Additionally, the infection affects the vesicles traffic, via RAS and Rab3a, that may modulate the entrance and survival of the pathogen inside the host cell vesicles. In addition, cytoskeleton-related proteins that mediate vesicle traffic could be regulated in the nucleus by the transcription factor SRE. Finally, we suggest that epigenetic regulation through CGBP and DMM51 may play a role by regulating methylation in CpG islands.

Although *A. baumannii* is regarded as an external pathogen, recent evidence suggests that it can survive, and even replicate intracellularly, in epithelial cells. (Figure 6). Our findings indicate that intracellular *A. baumannii* is able to deregulate the cell machinery to ensure its survival inside the host, similarly to other invasive bacteria, such as *Yersinia enterocolitica* (Figure S3, Supplementary Materials) [44]. The transcriptomic reaction of cells infected intracellularly by *A. baumannii* shows that they respond with an early activation of apoptosis and the stress response. Such responses are maintained during the infection process and even late after infection. Other responses, like GTPase activity, were observed early after infection, showing the need for cells to control cytoskeleton remodeling. Almost all the DEGs were downregulated late after infection, suggesting that *A. baumannii* is able to suppress the cell response and gain some control over cell functioning.

The control of cell functioning was also seen at the PPI level. Many of the up- and down-regulated genes were organized in protein complexes or as interacting proteins. Several complexes were upregulated early after infection, including those related to apoptosis, like TIAF1-HOXA5, and those linked to the stress response, like TXNIP-DDIT4. Most of these complexes were downregulated, including chemokines and chemokine receptors.

It is our conclusion that *A. baumannii* can survive and proliferate inside epithelial cells by modulating the host response. Its intracellular lifestyle has relevant implications for clinicians. Intracellular pathogens present inside host cells may well resist antibiotics better and evade the immune response; they are also able to proliferate by taking the resources they require from these cells. Even small loads of surviving bacteria after antibiotic treatment could promote a relapse of infection in patients, and this might explain the occurrence of repeated infections. A better understanding of the intracellular lifestyle of *A. baumannii* may help us to develop better treatments, identify useful biomarkers, and produce better clinical protocols to treat infectious diseases.

The intracellular existence of *A. baumannii* has been overlooked in the literature, but our research, despite its limitations, may pave the way to deciphering the mechanism that this pathogen uses to survive. New studies with complex cell-culture systems, including epithelial and innate immune cells, may help us to identify how the intracellular version of this bacteria survives. Moreover, in vivo models could further what is understood of the implications of intracellular survival after antibiotic treatment and how infection relapses occur.

## Data Availability

The data presented in this study are openly available at the Gene Expression Omnibus (GEO) database under code GSE161833.

## Supplementary Materials

The following are available online at https://www.mdpi.com/2076-2607/9/2/354/s1, Figure S1: Expanded representation of protein–protein interactions at 4 h post infection; Figure S2: Expanded representation of protein–protein interactions at 6 h post infection; Figure S3: Fold change correlation of DEGs identified during *A. baumanni* infection.
