# Supplementary material for: Time-Resolved Transcriptional Profiling of Epithelial Cells Infected by Intracellular Acinetobacter baumannii
Source: Microorganisms. 2021 Feb 11;9(2):354. doi: 10.3390/microorganisms9020354 (PMC7916935; doi:10.3390/microorganisms9020354)
Supplement: Supplementary file 1 [file microorganisms-09-00354-s001.pdf]

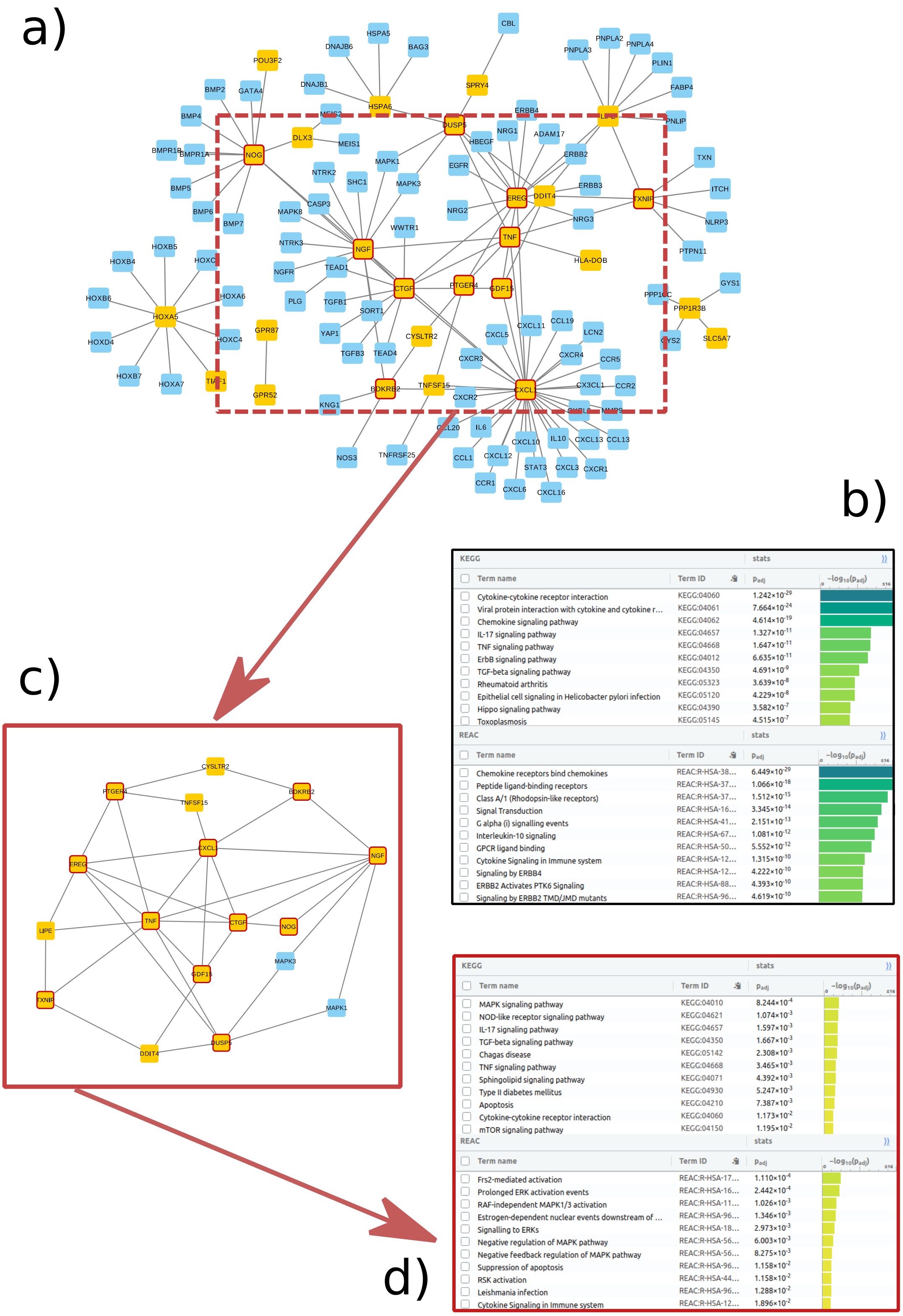

Figure S1. Extended Protein-Protein Interaction (PPI) network of our sorted DEGs at 4 h post-infection. Network diagrams are useful for visualizing hub proteins and their immediate connections. (**a**) Extended network, containing all first neighbors of detected DEGs. (**b**) GO terms associated to the extended network. (**c**) Densely packed clusters identified in the network and (**d**) the GO terms associated to these clusters.

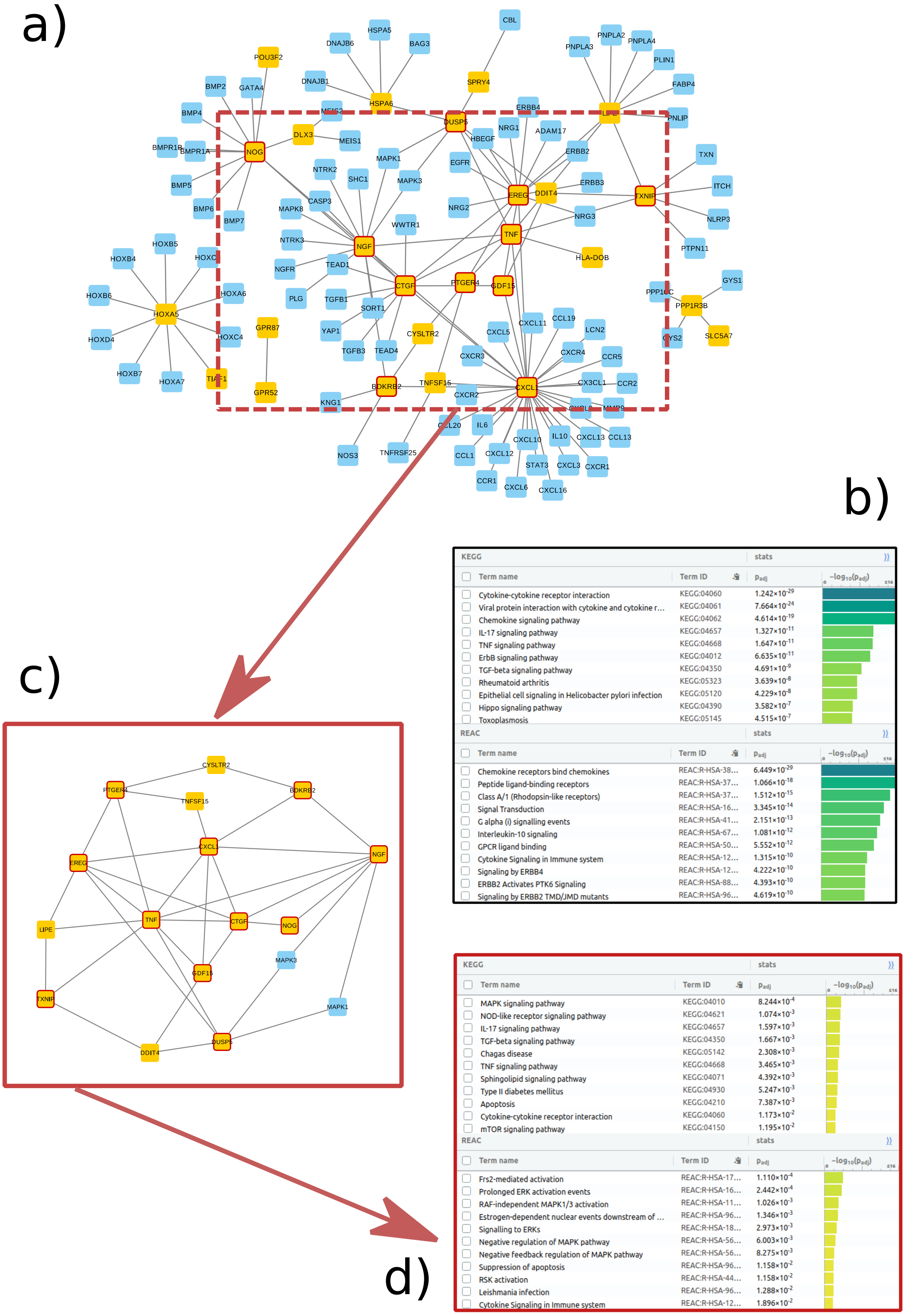

Figure S2. Extended Protein-Protein Interaction (PPI) network of our sorted DEGs at 6 h post-infection. Network diagrams are useful for visualizing hub proteins and their immediate connections. (**a**) Extended network, containing all first neighbors of detected DEGs. (**b**) GO terms associated to the extended network. (**c**) Densely packed clusters identified in the network and (**d**) the GO terms associated to these clusters.

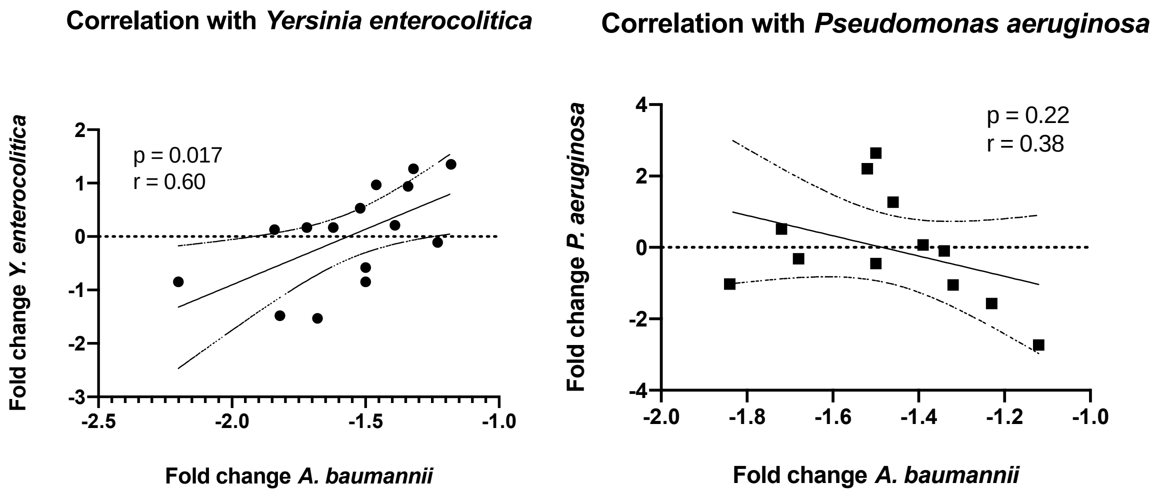

Figure S3. Fold change correlation of DEGs identified during *A. baumanni* infection with *Y. enterocolitica* (intracellular pathogen) and *P. aeruginosa* (extracellular pathogen). Fold change values for Y. enterocolitica and *P. aeruginosa* were obtained from the DualSeqDB database (http://www.tartaglialab.com/dualseq/) and *A. baumannii* were obtained from this study.
